# Chest X-ray findings in a large cohort of 1117 patients with SARS-CoV-2 infection: a multicenter study during COVID-19 outbreak in Italy

**DOI:** 10.1007/s11739-020-02561-3

**Published:** 2020-11-20

**Authors:** Valentina Vespro, Maria Carmela Andrisani, Stefano Fusco, Letizia Di Meglio, Guido Plensich, Alice Scarabelli, Elvira Stellato, Anna Maria Ierardi, Luigia Scudeller, Andrea Coppola, Andrea Gori, Antonio Pesenti, Giacomo Grasselli, Stefano Aliberti, Francesco Blasi, Chiara Villa, Sonia Ippolito, Barbara Pirrò, Guglielmo Damiani, Massimo Galli, Giuliano Rizzardini, Emanuele Catena, Matteo Agostino Orlandi, Sandro Magnani, Giuseppe Cipolla, Andrea Antonio Ianniello, Mario Petrillo, Genti Xhepa, Antonio Scamporrino, Alberto Cazzulani, Gianpaolo Carrafiello

**Affiliations:** 1 Department of Radiology, Fondazione IRCCS Ca’ Granda Ospedale Maggiore Policlinico, University of Milan, Milan, Italy; 2 Postgraduate School of Diagnostic and Interventional Radiology, University of Milan, Milan, Italy; 3 Scientific Direction, Clinical Epidemiology and Biostatistics, Fondazione IRCCS Ca’ Granda Ospedale Maggiore Policlinico, Milan, Italy; 4 Department of Radiology, Università degli Studi dell’Insubria, Varese, Italy; 5 Infectious Diseases Unit, Fondazione IRCCS Ca’ Granda Ospedale Maggiore Policlinico, University of Milan, Milan, Italy; 6 Department of Anesthesiology and Intensive Care Unit, Fondazione IRCCS Ca’ Granda Ospedale Maggiore Policlinico, University of Milan, Milan, Italy; 7 Department of Pathophysiology and Transplantation, University of Milan, Milan, Italy; 8 Department of Radiology, Luigi Sacco Hospital, ASST Fatebenefratelli Sacco, University of Milan, Milan, Italy; 9 Division of Infectious Diseases, Luigi Sacco Hospital, ASST Fatebenefratelli Sacco, University of Milan, Milan, Italy; 10 Department of Anesthesiology and Intensive Care Unit, Luigi Sacco Hospital, ASST Fatebenefratelli Sacco, University of Milan, Milan, Italy; 11 Department of Radiology, ASST Lodi, Ospedale Maggiore di Lodi, Lodi, Italy; 12 Department of Respiratory Diseases, ASST Lodi, Ospedale Maggiore di Lodi, Lodi, Italy; 13 Department of Radiology, ASST Rhodense, Garbagnate Milanese, Milan, Italy; 14 CERBA HealthCare Italia, Cologno Monzese, Monza Brianza Italy; 15 Department of Health Science, University of Milan, Milan, Italy

**Keywords:** COVID-19, Radiography, Thoracic, Diagnosis, Pandemics

## Abstract

To describe radiographic key patterns on Chest X-ray (CXR) in patients with SARS-CoV-2 infection, assessing the prevalence of radiographic signs of interstitial pneumonia. To evaluate pattern variation between a baseline and a follow-up CXR. 1117 patients tested positive for SARS-CoV-2 infection were retrospectively enrolled from four centers in Lombardy region. All patients underwent a CXR at presentation. Follow-up CXR was performed when clinically indicated. Two radiologists in each center reviewed images and classified them as suggestive or not for interstitial pneumonia, recording the presence of ground-glass opacity (GGO), reticular pattern or consolidation and their distribution. Pearson’s *χ*^2^ test for categorical variables and McNemar test (*χ*^2^ for paired data) were performed. Patients mean age 63.3 years, 767 were males (65.5%). The main result is the large proportion of positive CXR in COVID-19 patients. Baseline CXR was positive in 940 patients (80.3%), with significant differences in age and sex distribution between patients with positive and negative CXR. 382 patients underwent a follow-up CXR. The most frequent pattern on baseline CXR was the GGO (66.1%), on follow-up was consolidation (53.4%). The most common distributions were peripheral and middle-lower lung zone. We described key-patterns and their distribution on CXR in a large cohort of COVID-19 patients: GGO was the most frequent finding on baseline CXR, while we found an increase in the proportion of lung consolidation on follow-up CXR. CXR proved to be a reliable tool in our cohort obtaining positive results in 80.3% of the baseline cases.

## Introduction

In late December 2019, local health authorities reported clusters of patients with pneumonia of unknown etiology, epidemiologically linked to a seafood market in Wuhan, Hubei Province, China [1].

A surveillance mechanism for “pneumonia of unknown etiology” was established with the aim of allowing timely identification of novel pathogens; the pathogen, a novel coronavirus (SARS-CoV-2), was identified. On 30 January 2020, the World Health Organization (WHO) declared that COVID-19 is a “public-health emergency of international concern” [2].

The pandemic has escalated rapidly; the first person-to-person transmission in Italy was reported on Feb 21st, 2020 [3]. WHO data as of April 25th, 2020, reported 192,994 confirmed cases in Italy, with 25,969 deaths and a lethality rate of 13.5% [4].

The diagnosis and treatment program (7th version) published by the National Health Commission of the People’s Republic of China no longer considers the radiologic features of lung involvement a diagnostic criteria for COVID-19 [5]. Early discussions suggested that computed tomography (CT) should be the preferred modality for diagnosis of COVID-19, due to its higher sensitivity. However, the use of CT for COVID-19 diagnosis is controversial [6, 7].

In addition, in most hospitals, utilization of CT room for every patient with COVID-19 infection ascertained or suspected should be not easily manageable in terms of staff commitment, CT room workflow and disinfection procedures. Like any procedure performed on highly infective patients, it’s mandatory to guarantee the effectiveness of the isolation itself, the safety of medical staff at risk.

Moreover, with the dramatic trend described above, early diagnosis of this newly emerging and life-threatening infection is crucial for the management of those patients, and therefore diagnostic procedures should be limited to the essential ones.

So far, radiological literature has primarily focused on CT findings, and a detailed overview of CXR appearance in relation to the disease is still poor. Therefore, the primary aim of our retrospective multicentric study was to describe the radiographic key patterns on Chest X-Ray (CXR) in patients with SARS-CoV-2 infection. In addition, we aimed at assessing the prevalence of individual radiographic signs of COVID-19 pneumonia among all the patients tested positive for SARS-CoV-2. Also, we compared patients with normal CXR vs those with at least one abnormal finding, in terms of demographic and clinical variables; finally, we estimated pattern variation between a baseline CXR and a follow-up CXR, to assess the utility of CXR in supporting the management of COVID-19 pneumonia.

## Materials and methods

### Setting

Four hospitals in Lombardy region participated to the study: three located in Milan area (ASST Fatebenefratelli Ospedale Luigi Sacco, Fondazione IRCCS Ca’ Granda Ospedale Maggiore Policlinico, ASST Rhodense—Presidio Ospedaliero di Garbagnate Milanese), and one in Lodi (ASST Lodi—Ospedale Maggiore di Lodi).

This retrospective cross-sectional study, with a longitudinal component, was conducted in accordance with the principles of the Declaration of Helsinki. Each institutional review board approved the study according to General Data Protection Regulation (GDPR).

### Patients

All consecutive adult (> 18 years) patients referred to the Emergency Department (ED), with laboratory-identified SARS-CoV-2 infection by real time Reverse Transcription-Polymerase Chain Reaction (RT-PCR), were enrolled between February 22nd, 2020, and March 18th, 2020. All patients had at least one CXR at presentation.

The following clinical variables were extracted from patient’s charts: age, sex, exposure history, comorbid conditions, symptoms.

A repeat CXR was performed in the admission ward, when clinically indicated by the attending physician.

### RT-PCR test

Nasopharyngeal and oropharyngeal specimens collected with synthetic fiber swabs (manufactured by Copan) were laboratory tested with real time RT-PCR to detect SARS-CoV-2 nucleotides.

The real time RT-PCR Tests were performed using GeneFinder™ COVID-19 Plus Real*Amp* Kit manufactured by OSANG Healtcare (CE-IVD marked, fulfilling European Directive 98/79/EC) and Allplex™ 2019-nCoV Assay manufactured by Seegene (approved by Korean Food and Drug Administration).

### Imaging acquisition and interpretation

The majority of CXR were performed bedside with portable digital radiographic equipment, owing to the impossibility to move the patient and/or to avoid his transportation from isolation areas. In the other cases CXR were acquired with patient standing with standard two projections.

Different digital radiographic equipment has been used: Ysio Max system and MOBILETT Elara Max mobile system (Siemens Healthcare) [Hospital A], Adora System (NRT X-RAY A/S) and MAC mobile X-ray unit (General Medical Merate) [Hospital B], AXIOM Luminos dRF system (Siemens Healthcare) and FCR Go 2 portable system (Fujifilm) [Hospital C], FDR AcSelerate system and FDR Go PLUS portable system (Fujifilm) [Hospital D].

In each Hospital, two radiologists (with more than 10 years of experience in interpreting CXR imaging), reviewed all images, and classified CXR as suggestive or not suggestive for COVID-19 pneumonia. In case of disagreement, the same radiologists reviewed together the images to reach a consensus on the basis of the available radiological literature data.

The epidemiological history and clinical symptoms (cough, fever, weakness, etc.) were available for both readers.

The presence of lung parenchymal abnormalities on each CXR was recorded in accordance with the Glossary of Terms for Thoracic Imaging of the Fleischner Society and defined as: (a) ground glass opacity (GGO); (b) lung consolidation; (c) reticular pattern [8].

**Fig. 1:**
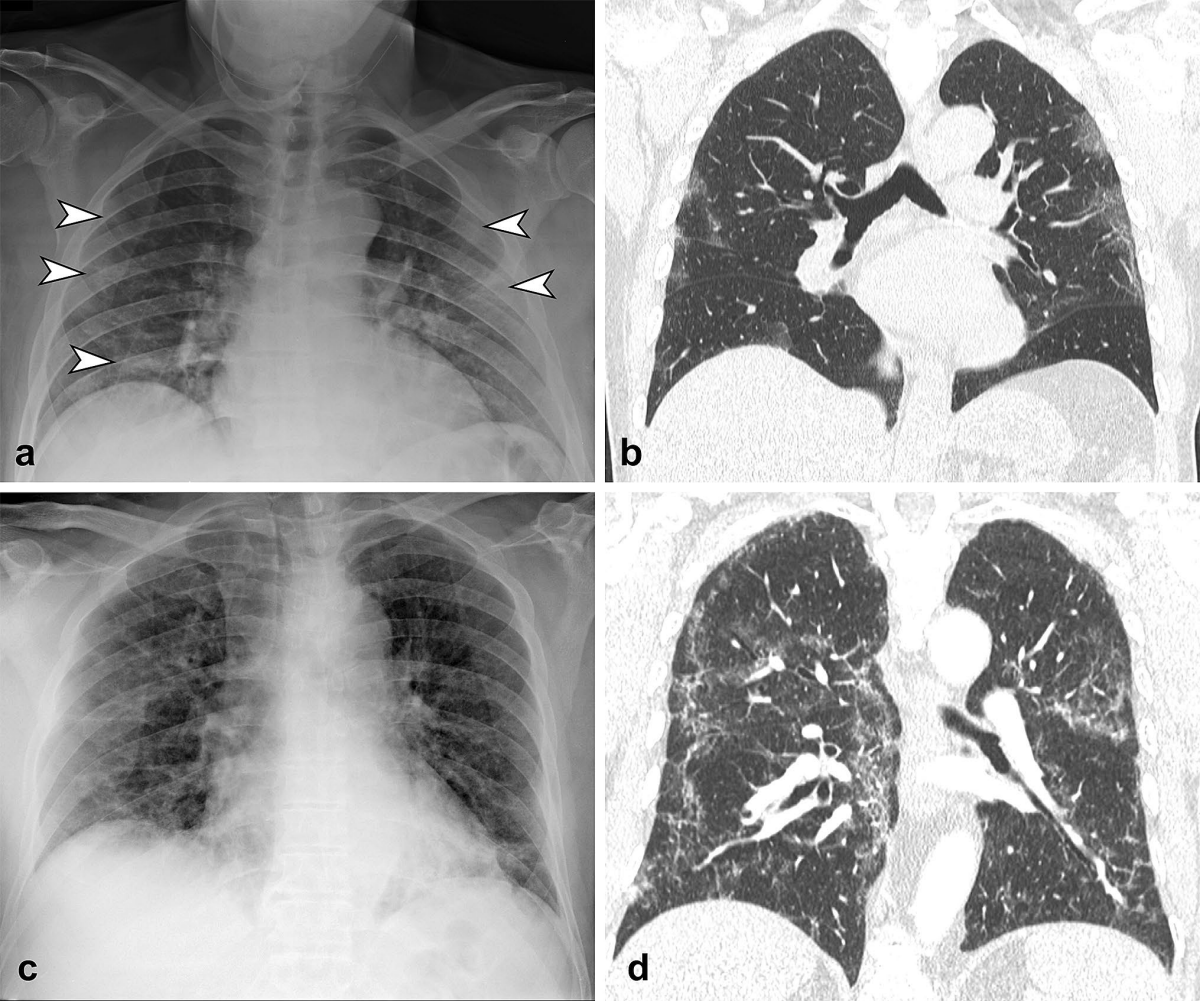
**a** CXR in antero-posterior view shows bilateral ground glass opacities (arrowheads) with peripheral distribution, involving middle-lower zone of the lungs. **b** Coronal reformatted CT image confirmed the CXR findings. **c** CXR in antero-posterior view shows bilateral reticular pattern with diffuse distribution on both axial and longitudinal plane. **d** Coronal reformatted CT image confirms the presence of diffuse interstitial involvement with reticular pattern

**Fig. 2:**
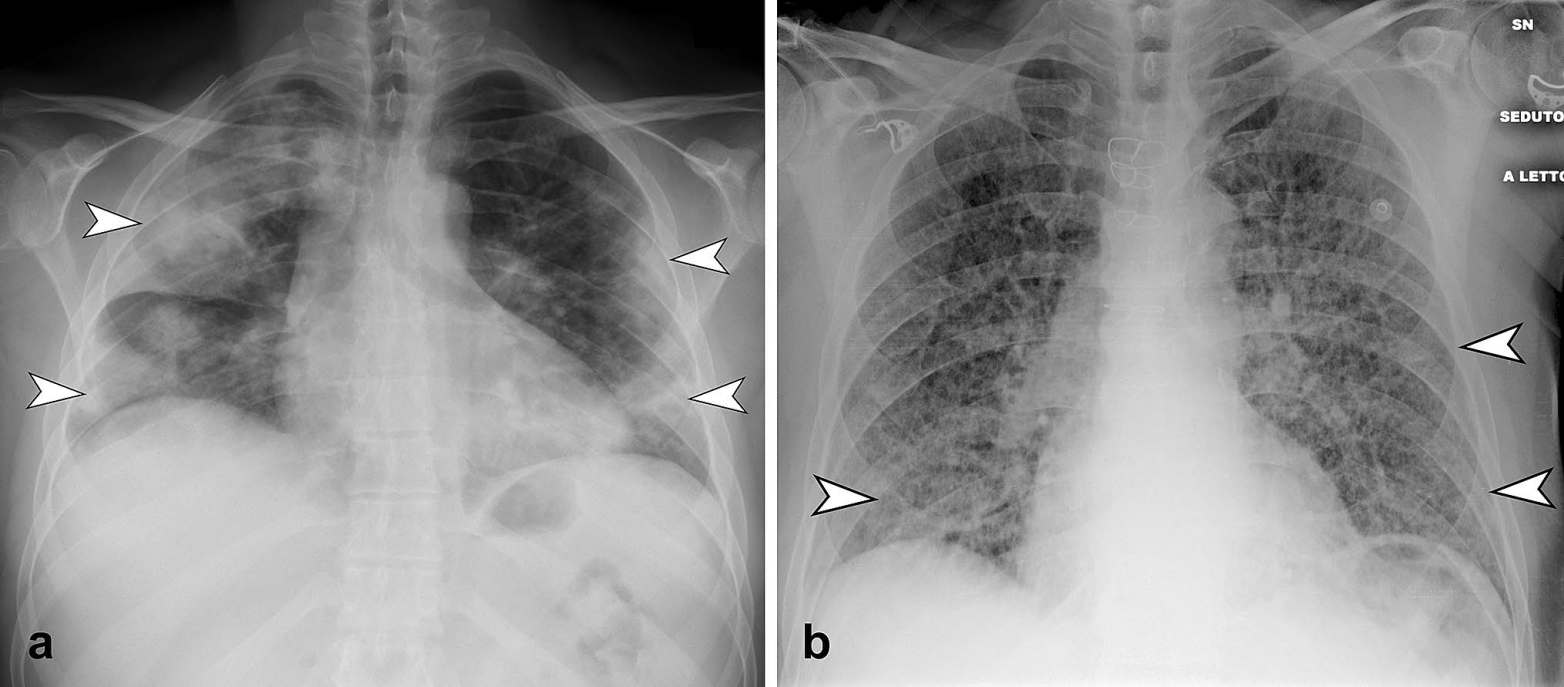
**a** CXR in postero-anterior view shows bilateral multifocal consolidation (arrowheads), with greater involvement of the right lung. Consolidation present peripheral predominant distribution. **b** CXR in antero-posterior view shows the presence of diffuse bilateral reticular pattern. Note the compresence of bilateral subtle GGO (arrowheads) with peripheral and middle-lower predominant distribution

GGO is defined as an area of hazy increased lung opacity, less opaque than consolidation, within which margins of pulmonary vessels may be indistinct (Fig. 1a, b). Consolidation is defined as a homogeneous increase in pulmonary parenchymal attenuation that obscures the margins of vessels and airway walls (Fig. 2a). Reticular pattern is defined as a collection of innumerable small linear opacities that, by summation, produce an appearance resembling a net (Fig. 1c, d, Fig. 2b).

Patients were considered to have findings suggestive for COVID-19 pneumonia if they had at least one of the above-mentioned features.

The distribution of the abnormal findings was recorded considering three criteria: (a) laterality (unilateral or bilateral); (b) axial distribution (central and/or peripheral); (c) longitudinal distribution (superior and/or middle and/or inferior).

Associated findings such as pleural effusion, pneumothorax and pneumomediastinum, were also recorded.

In addition, on follow-up CXR radiologists evaluated the evolution of the radiographic findings describing if any improvement, a worsening or no significant changes occurred.

### Statistical analysis

The study sample size was targeted at least 1000 patients, to be able to estimate proportions with precision at least 3.2%. Descriptive statistics were produced for demographic, clinical and laboratory characteristics of cases. Mean and standard deviation (SD) are presented for normally distributed variables, and median and interquartile range (IQR) for non-normally distributed variables, number and percentages for categorical variables. Groups were compared with parametric or nonparametric tests, according to data distribution, for continuous variables, and with Pearson’s *χ*^2^ test (Fisher exact test where appropriate) for categorical variables. Differences between first and second CXR were tested by means of the McNemar test (*χ*^2^ for paired data). In all cases, 2-tailed tests were used. *P* value significance cut-off was 0.05. Stata computer software version 16.0 (Stata Corporation, 4905 Lakeway Drive, College Station, Texas 77845, USA) was used for statistical analysis.

## Results

The 1171 patients enrolled in our study had a mean age of 63.3 years (SD 15.9; range 18–96). There were 767 males (65.5%) (Table 1). Fever was the most frequent symptom (79.9%), while cough was present in 50.7% of the patients; 54 patients (4.6%) were asymptomatic. The most common comorbidities were arterial hypertension (31.2%), diabetes (13.5%) and cardiovascular diseases (13.4%).

**Table 1:** Patient clinical characteristics

	All patients (*n* = 1171)	Patients with positive baseline CXR (*n* = 940)	Patients with negative baseline CXR (*n* = 231)	*p* value
Characteristics				
Mean age (years)	63.3 (SD 15.9)	65 (SD 14.7)	56.6 (SD 18.4)	0.001
Sex				
Male	767 (65.5%)	634 (67.5%)	133 (57.6%)	0.005
Female	404 (34.5%)	306 (32.55%)	98 (42.4%)	0.005
Symptoms				
Fever	935 (79.9%)	739 (78.6%)	196 (84.9%)	< 0.001
Cough	594 (50.7%)	476 (50.6%)	118 (51.1%)	< 0.001
Dyspnea	411 (35.1%)	361 (38.4%)	50 (21.7%)	< 0.001
Sore throat	25 (2.1%)	10 (1%)	15 (6.5%)	–
Diarrhea	44 (3.8%)	38 (4%)	6 (2.6%)	–
Vomiting	24 (2%)	21 (2.2%)	3 (1.3%)	–
Headache	18 (1.5%)	12 (1.3%)	6 (2.6%)	–
Syncope	32 (2.7%)	25 (2.7%)	7 (3%)	–
Asymptomatic	54 (4.6%)	38 (4%)	16 (6.9%)	< 0.001
Comorbidities				
Any	692 (59.1%)	576 (61.3%)	116 (50.2%)	–
Hypertension	365 (31.2%)	313 (33.3%)	52 (22.5%)	–
Diabetes	158 (13.5%)	126 (13.4%)	32 (13.9%)	–
Cardiovascular disease	157 (13.4%)	129 (13.7%)	28 (12.1%)	–
Pulmonary disease	106 (8.8%)	78 (8.3%)	28 (12.1%)	0.001
Malignancy	60 (5.1%)	58 (6.2%)	2 (0.9%)	–

CXR showed lung abnormalities in 940 patients (80.3%). There were significant differences in age distribution between patients with positive (mean age: 65 years [SD 14.7]) and negative (56.6 years [SD 18.4]—[*p* value < 0.001]) radiographic findings. Sex distribution also presented significant differences (positive CXR: 67.5% males—32.5% females; negative CXR: 57.6% males—42.4% females—[*p* value = 0.005]).

The prevalence of fever and/or cough was similar among patients with positive CXR findings and patients with negative CXR, while dyspnea was present in 38.4% of patients with positive radiographic findings and in 21.7% of patients with negative radiographic findings (*p* value < 0.001).

### Radiographic findings on baseline CXR

Of the 940 positive chest radiographs, 638 (67.9%) showed the presence of a single pattern, while 273 (29%) had the compresence of two patterns and 29 (3.1%) presented three patterns.

The most frequent radiographic pattern, either isolated or combined, was the GGO (621/940, 66.1%), followed by reticular pattern (426/940, 45.3%) and consolidation (224/940, 23.8%).

Peripheral (524/940, 55.8%) and middle-lower zone distribution (331/940, 33.1%) were the most common locations, and the 73.9% (695/940) had bilateral involvement.

Pleural effusion was described only in 7 cases.

The frequency of the three patterns and their distribution are listed in Table 2.

**Table 2:** Findings on positive baseline CXR (*n* = 940)

Patterns (alone or combined)	
GGO	621 (66.1%)
Reticular pattern	426 (45.3%)
Consolidation/s	224 (23.8%)
Number of patterns present	
One	638 (67.9%)
Two	273 (29%)
Three	29 (3.1%)
Pattern combinations	
GGO	363 (38.6%)
GGO + reticular pattern	184 (19.6%)
Reticular pattern	169 (18%)
Consolidations	90 (9.6%)
GGO + consolidations	45 (4.8%)
Reticular pattern + consolidations	40 (4.3%)
GGO + reticular pattern + consolidations	29 (3.1%)
Single consolidation	16 (1.7%)
Single consolidation + reticular pattern	4 (0.4%)
Axial distribution	
Peripheral	524 (55.7%)
Central	311 (33.1%)
Diffuse (Central + peripheral)	105 (11.2%)
Longitudinal distribution	
Middle + inferior	375 (39.9%)
Inferior	208 (22.1%)
Superior + middle	78 (8.3%)
Middle	77 (8.2%)
Superior	46 (4.9%)
Superior + inferior	30 (3.2%)
Diffuse (Superior + middle + inferior)	126 (13.4%)
Laterality	
Unilateral	245 (26.1%)
Bilateral	695 (73.9%)

### Radiographic findings on follow-up CXR

382 patients underwent a follow-up CXR. The median time between the two examinations was 4 days (IQR 3; range 1–17).

In 351 patients the first CXR examinations yielded positive results, confirmed on the second CXR.

The remaining 31 patients had normal findings on the first examination: the subsequent CXR showed radiographic abnormalities in 26 cases, while confirmed normal findings in 5 cases. Therefore, the overall prevalence of positive follow-up CXR was 98.7% (377/382).

In 262/382 cases (68.6%) we noticed a worsening of the imaging findings, 73/382 (19.1%) CXR were not significantly changed, while in 47/382 (12.3%), cases there was an improvement.

**Fig. 3:**
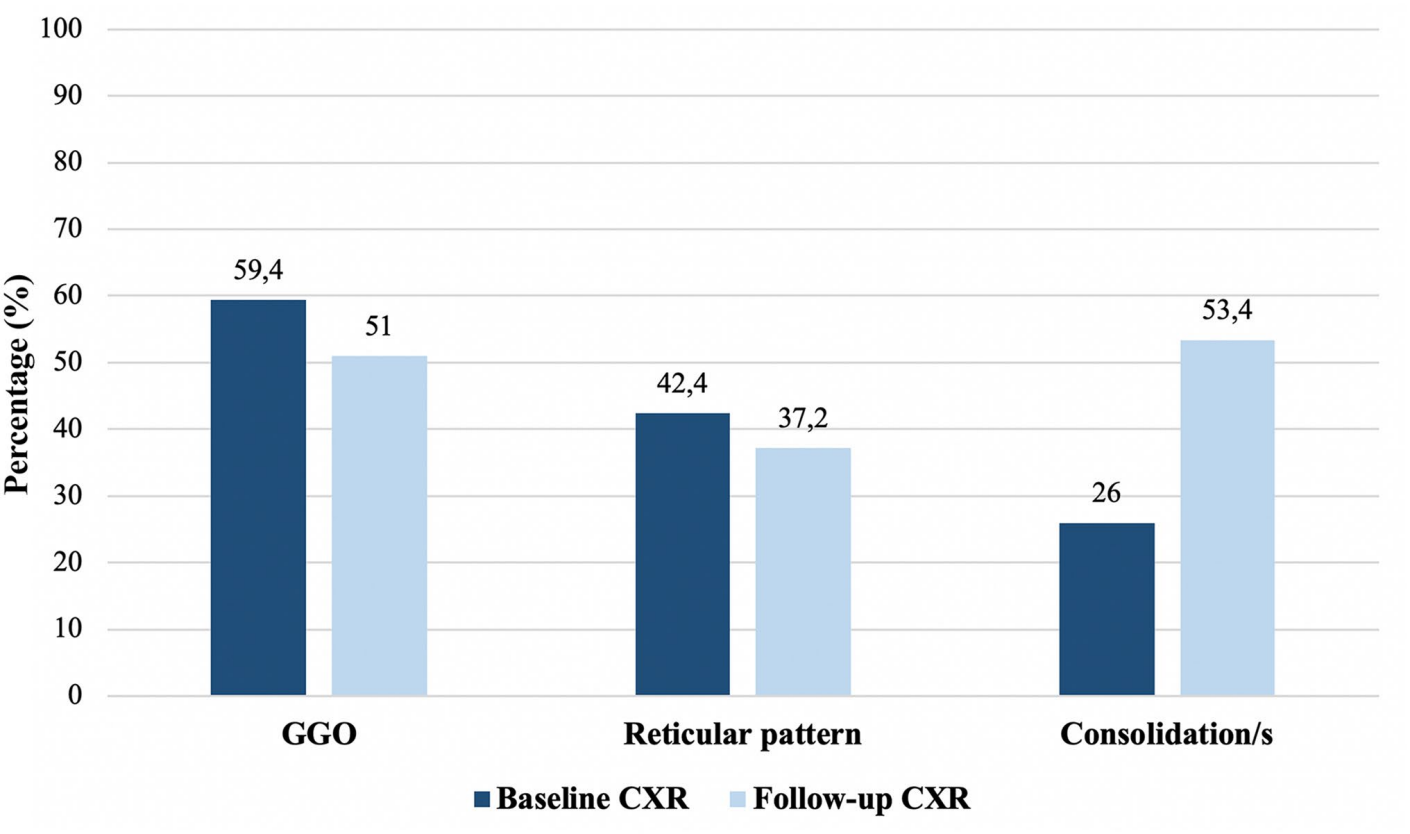
Frequency of radiographic patterns on baseline and follow-up CXR (*n* = 382)

The prevalence of the key patterns in the follow-up group changed as follows: consolidation was described in the 26% (99/382) of cases on the baseline CXR, while was present in the 53.4% (204/382) on the follow-up CXR; on the other hand, the prevalence of GGO and reticular pattern decreased on the follow-up CXR, passing from 59.4% (227/382) to 51% (195/382) and from 42.4% (162/382) to 37.2% (142/382), respectively (Fig. 3).

As seen above, the prevalence of consolidation in the follow-up group, increased significantly (from 26 to 53.4%—*p* value < 0.001); furthermore, 116 patients with no consolidation on the baseline CXR showed consolidation on the follow-up CXR, while only in 12 cases consolidation was seen on the first CXR and was no longer present at the follow-up.

Peripheral (48.1%) and middle-lower zone distribution (40.2%) remained the most common locations of the lung abnormalities, but we observed a greater involvement of the lung parenchyma both on axial and longitudinal distribution, in particular central-peripheral distribution was present in the 47.6% of patients (in contrast with 39.6% on the baseline CXR) and superior–middle–inferior distribution was described in the 27.9% of patients (in contrast with 16.8% on the baseline CXR). Moreover, the percentage of bilateral involvement of the lung increased from 76.6 to 87.7%.

Other findings recorded were pleural effusion (6/382), pneumothorax (2/382) and pneumomediastinum (2/382).

The comparison between the findings on follow-up and baseline CXR is listed in Table 3.

**Table 3:** Comparison of findings in patient with baseline and follow-up CXR (*n* = 382)

	Baseline CXR	Follow-up CXR	*p* value
Positive	351 (91.2%)	377 (98.7%)	–
No abnormalities	31 (8.8%)	5 (1.3%)	–
Patterns (alone or combined)			
GGO	227 (59.4%)	195 (51%)	< 0.001
Reticular pattern	162 (42.4%)	142 (37.2%)	0.0181
Consolidation/s	99 (26%)	204 (53.4%)	0.0039
Axial distribution^a^			< 0.001
Peripheral	181 (51.6%)	169 (48.1%)	
Central	32 (9.1%)	15 (4.3%)	
Diffuse (Central + Peripheral)	138 (76.6%)	167 (87.7%)	
Longitudinal distribution^a^			< 0.001
Middle–inferior	139 (39.6%)	141 (40.2%)	
Inferior	60 (17.1%)	37 (10.5%)	
Superior–middle	32 (9.1%)	33 (9.4%)	
Middle	29 (8.3%)	22 (6.3%)	
Superior	21 (6%)	7 (2%)	
Superior–inferior	11 (3.1%)	13 (3.7%)	
Diffuse (superior–middle–inferior)	59 (16.8%)	98 (27.9%)	
Laterality^a^			< 0.001
Unilateral	82 (23.4%)	43 (12.3%)	
Bilateral	269 (76.6%)	308 (87.7%)	

*N.B.* the McNemar test for paired data was used (please see statistical methods)

^a^Percentages refer to patients with positive baseline CXR (*n* = 351)

## Discussion

The main result of this study is the large proportion of positive CXR in patients with SARS-CoV-2 infection admitted to ED.

The most frequent lung abnormality detected on CXR was the GGO, present in 66.1% of our positive baseline CXR, confirming the data of the recent literature on both CXR and CT findings [6, 9–12].

Moreover, in line with the common CT findings of recent studies, the most frequent locations of lung abnormalities were the peripheral and middle-lower zone distribution, suggesting that the disease, may initially involve the parenchyma distal to the secondary lobule [13]. The bilateral involvement was also prevalent both in the baseline CXR (73.9%) and in the follow-up CXR (85.9%), consistent with other studies [6, 9, 14–16].

The reassessment of 382 patients with a follow-up CXR, has revealed an increase in the proportion of lung consolidation on positive CXR from about one fourth to over half. According to recent literature, this reflects the spectrum of diffuse alveolar damage (DAD) and/or organizing pneumonia, which tends to arise later in the disease course [14, 17, 18]. Furthermore, we were able to assess a greater extent of lung parenchymal involvement on follow-up radiographs. Since the repeat CXR was requested at discretion of the attending physician in case of suspected clinical worsening, these results suggest that there is an association between CXR findings evolution and the clinical progression of the disease [11, 14].

The vast majority of our patients were symptomatic and more than three quarters of our baseline CXR were positive. This data can be explained by the fact that, as opposed to what happened in Wuhan (China), where patients were encouraged to present to the hospital early in the course of their disease, in Italy the local authorities invited patients to stay at home until they would have experienced advanced symptoms [19, 20].

In such context, CXR often shows lung abnormalities and can be used as a first line assessment, while it is known that CXR has a low sensitivity in detecting early manifestations of COVID-19. Moreover it is important to be aware that even chest CT has been reported to demonstrate no abnormalities in the first 3 days of symptoms in 56% of patients [21].

It is common knowledge that CXR has a lower sensitivity and specificity than CT. Nevertheless, due to the delicate management of high infectious patients, CXR has the considerable advantage of being conducted in the Biocontainment Unit (BU) or in isolating areas by mean of a portable digital radiographic equipment, thus reducing the risk of infection for healthcare workers and other patients. On the other hand, the use of a CT room for every patient with an ascertained or suspected SARS-CoV-2 infection must be carefully managed in terms of staff commitment, CT room workflow, disinfection procedures and consumption of personal protection equipment (PPE) [22]. Moreover, CXR also reduces the risk of radiation exposure to the patient.

As stated by the Multinational Consensus from the Fleischner Society, the choice of imaging modality should be subjected to the judgement of clinical teams taking into account the different properties of CXR and CT, the local resources and expertise [20].

Strengths of our study are the large sample size, unbiased representation of patients admitted to ED in our Region. However, our study presents several limitations. First of all, only 32.6% of our patients underwent a follow-up CXR. Secondly, we have limited clinical data due to the worldwide emergency situation; in particular we could not obtain information timing of symptoms onset. Thirdly, the interval between the baseline and the follow-up CXR is quite variable; also, we lack data about final patient outcomes. Moreover, we only included patients with CXR; however, the number of RT-PCR positive patients lacking CXR is estimated < 1%, and this is unlikely to have caused bias. Finally, a control group with negative RT-PCR testing is lacking; however, we aimed at representing current clinical practice, where only patients with positive RT-PCR testing are diagnosed as proven infections.

It would be of strong clinical value to extend the study to all patients who access to ED with suspected SARS-CoV-2 infection, to evaluate the role of CXR in distinguishing patients with or without COVID-19.

In addition, it would be interesting to analyze the potential prognostic value of baseline CXR in predicting clinical outcome.

In conclusion, we have managed to describe the CXR key-patterns of COVID-19 and their distribution in a large cohort of patients during the peak of the COVID-19 outbreak in Italy. GGO was the most frequent finding on the baseline CXR, while we found an increase in the proportion of lung consolidation on the follow-up CXR. Despite being less sensitive than CT, CXR proved to be a reliable tool in our cohort obtaining positive results in 80.3% of the baseline cases.

Therefore, we believe that, in this specific epidemiological context, CXR should be the first line imaging technique. CT should be reserved to selected cases, e.g., in ruling out potential causes of acute symptoms worsening.

## Abbreviations

COVID-19: Coronavirus disease 2019
CT: Computed tomography
CXR: Chest X-ray
ED: Emergency department
GGO: Ground glass opacity
RT-PCR: Reverse transcription polymerase chain reaction
SARS-CoV-2: Severe acute respiratory syndrome-coronavirus-2
